# A dendritic cell-like biomimetic nanoparticle enhances T cell activation for breast cancer immunotherapy†

**DOI:** 10.1039/d1sc03525h

**Published:** 2021-11-25

**Authors:** Yanhua Li, Kun Tang, Xia Zhang, Wei Pan, Na Li, Bo Tang

**Affiliations:** College of Chemistry, Chemical Engineering and Materials Science, Key Laboratory of Molecular and Nano Probes, Ministry of Education, Collaborative Innovation Center of Functionalized Probes for Chemical Imaging in Universities of Shandong, Institute of Molecular and Nano Science, Shandong Normal University Jinan 250014 P. R. China lina@sdnu.edu.cn tangb@sdnu.edu.cn

## Abstract

Cancer immunotherapy has remarkably improved the therapeutic effect of melanoma and non-small cell lung cancer in the clinic. Nevertheless, it showed disappointing clinical outcomes for treating immunosuppressive tumors, wherein aggressive T cells are rather limited in tumor sites. Therefore, regulating the behavior of T cells in tumor sites to increase their attack ability for suppressing the immunosuppressive tumor is highly desirable. Inspiringly, we designed a dendritic cell-like biomimetic nanoparticle (DMSNs^3^@HA) to regulate the behavior of T cells for improving the immunotherapy effect against immunosuppressive tumors. In this work, anti-CD3 and anti-CD28 were responsible for mimicking dendritic cells to activate T cells, and anti-PD-1 for blocking the pathway of PD-1/PD-L1 to break the immune “brake”, which synergistically regulated the behavior of T cells to attack cancer cells. Experimental results indicated that DMSNs^3^@HA can effectively activate T cells and improve their immune response to significantly inhibit the growth of breast cancer. Moreover, it also proved that T cell activation combining immune checkpoint blocking induced the “1 + 1 >2” immunotherapy effect against immunosuppressive tumors. We expect that this strategy will provide new insights into tumor immunotherapy by modulating T cell behavior.

## Abstract 2

A dendritic cell-like biomimetic nanoparticle has been designed to regulate the behavior of T cells for improving the immunotherapy effect against immunosuppressive tumors.

## Graphical abstract

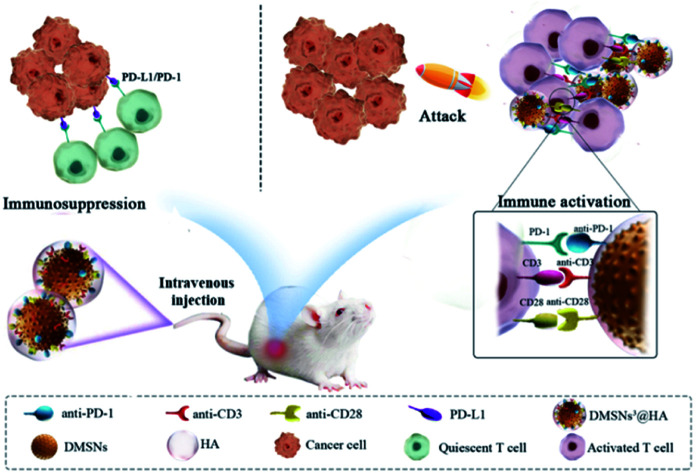

## Introduction

Cancer immunotherapy has become a revolutionizing treatment method after traditional surgery, chemotherapy and radiotherapy in recent years and has reformed the mindset from the direct destruction of cancer cells to the rejuvenation of the host's anti-tumor immune response for recognizing and attacking the cells.^1–5^ As one of the most critical immune cells in the immune system, T cells are the body's “fighters” against tumors and can efficiently kill tumor cells after activation.^6–10^ Immune checkpoint blocking therapies for the reversal of T cell exhaustion have markedly improved the treatment of melanoma and non-small cell lung cancer in the clinic.^11–16^ Notwithstanding, the anti-tumor immune responses are usually insufficient to control immunosuppressive tumors (such as breast cancer) by such methods.^17^ One of the intrinsic limitations is the lack of aggressive T cells in the tumor microenvironment of immunosuppressive tumors.^18–20^ Therefore, it is desirable to develop strategies that can improve the immune response of T cells in force at the tumor site to suppress the growth of immunosuppressive tumors.

Dendritic cells (DCs), known for their “tree-like” or dendritic shapes, are the most powerful professional antigen-presenting cells in the body, which play a vitally important role in initiating, regulating, and maintaining the immune response.^21,22^ DCs can evoke T cell activation by cellular interactions between the co-stimulating receptor and CD28 to stimulate the immune response.^23^ However, the expression of the co-stimulating receptor on DCs requires the stimulus of the immune vaccine or adjuvant, which introduces complex operation and may have great side effects.^24,25^ Biomimetic nanoparticle mimicking dendritic cells for activating T cells would favor the immune response for cancer immunotherapy.

**Scheme 1:**
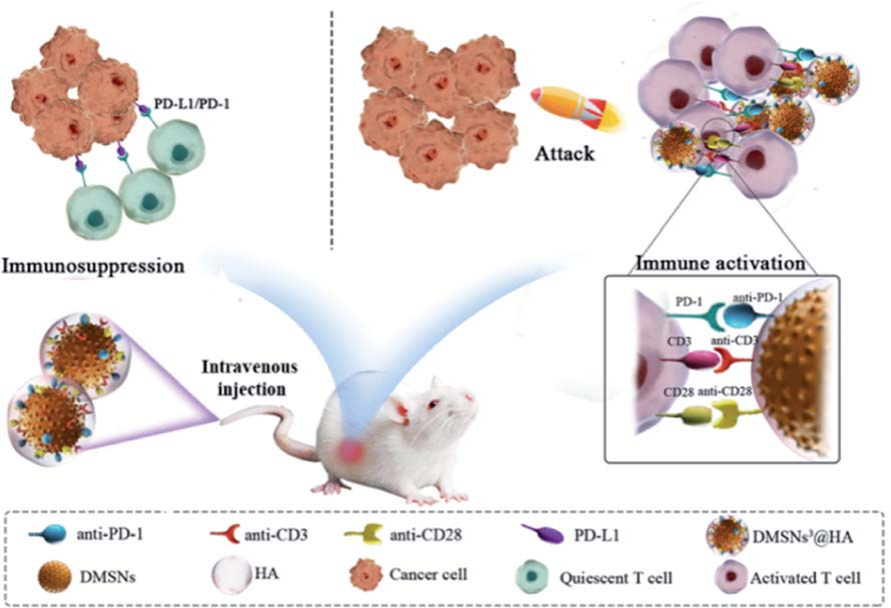
Schematic illustration of DMSNs^3^@HA for enhancing T cell activation and breast cancer immunotherapy.

In this regard, we designed a dendritic cell-like biomimetic nanoparticle that can simultaneously activate T cells and break the immune “brake” of T cells at the tumor site for improving the immune response of T cells and thus inhibiting the growth of immunosuppressive tumors (Scheme 1). Dendritic mesoporous silica nanoparticles (DMSNs) are an excellent candidate for preparing biomimetic nanoparticles due to their good biocompatibility, large specific surface area, easy modification and similar function to immune adjuvants. Carboxylated DMSNs were synthesized and covalently modified with functional antibodies (anti-CD3 and anti-CD28 for activating T cells and anti-PD-1 for breaking the immune “brake” of T cells) by the reaction of amino and carboxyl groups (DMSNs^3^). An intact dendritic cell-like biomimetic nanoparticle (DMSNs^3^@HA) was modified with hyaluronic acid (HA) to gain the ability to target tumor tissues. When intravenously injected, DMSNs^3^@HA were enriched at the tumor site by binding to CD44 overexpressed on the cancer cell membrane. And anti-CD3 and anti-CD28 functioned as costimulatory molecules to activate T cells by acting with CD28 on the membrane of T cells. Furthermore, anti-PD-1 could break the immune “brake” by interrupting the immune checkpoint PD-1/PD-L1. The immunotherapeutic effect of immunosuppressive tumors can be significantly improved by the mobilization of T cells.

## Results and discussion

### Synthesis and characterization of DMSNs^3^@HA

**Fig. 1:**
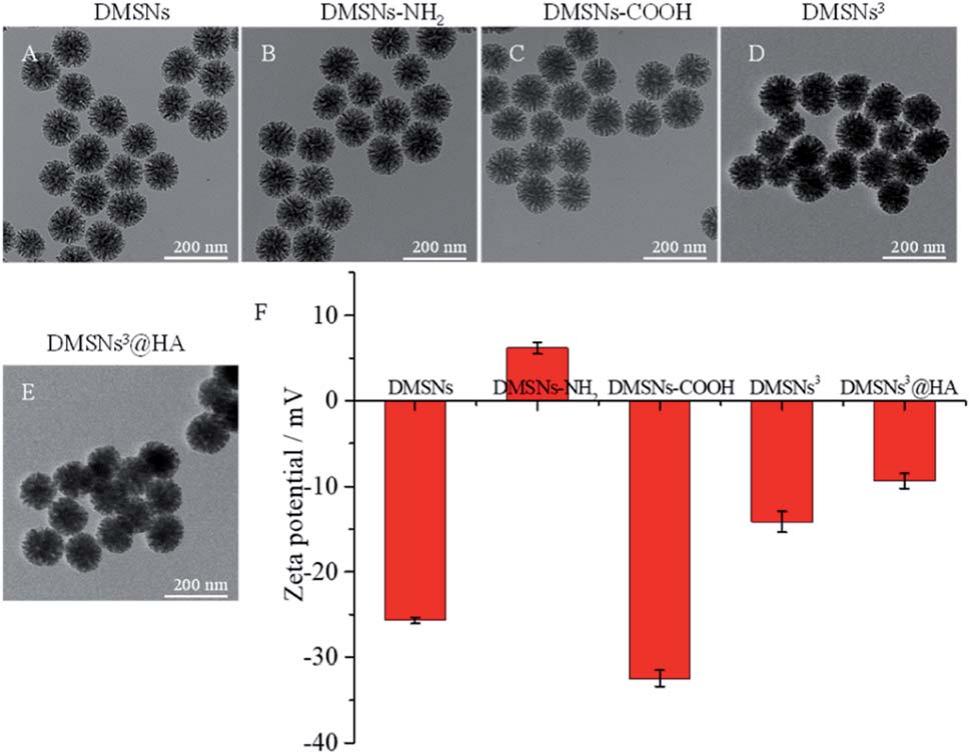
TEM images of DMSNs (A); DMSNs-NH_2_ (B); DMSNs-COOH (C); DMSNs^3^ (D); DMSNs^3^@HA (E). Zeta potentials of materials (F).

DMSNs were synthesized by means of methods reported previously with some modifications.^26,27^ Transmission electron microscopy (TEM) images in Fig. 1A explicitly revealed that DMSNs represented a uniform dendritic morphology with a size of 120 ± 10 nm. The hydrodynamic diameter of DMSNs was ∼160 nm in water obtained by the dynamic light scattering (DLS) assay (Fig. S1†). Then the DMSNs were modified with amino and carboxyl groups step by step to form DMSNs-NH_2_ and DMSNs-COOH. TEM images in Fig. 1B and C revealed that the modification of amino and carboxyl groups had no effect on the morphology of DMSNs.

Ninhydrin is a common reagent for detecting amines. When reacted with primary amines at the boil, it produces the Ruhemann substance with a dark blue or purple color. When reacted with primary amines plus amido bonds (CO–NH), such as glutamine, it produces a brown substance.^28,29^ Ninhydrin assay data exhibited an achromatic or yellowish color for the supernatant liquid of centrifuged DMSNs-NH_2_ and a bluish violet color for the precipitate of DMSNs-NH_2_, which verified the successful modification of amino groups on the DMSNs (Fig. S2†). The Fourier transform infrared spectroscopy (FT-IR) data of DMSNs, DMSNs-NH_2_ and DMSNs-COOH showed that the DMSNs were modified with amino and carboxyl groups successively (Fig. S3†). The quantification of amino and carboxyl groups of the DMSNs was 1.27 μmol mg^−1^ and 0.77 μmol mg^−1^ DMSNs calculated by thermogravimetric analysis (TGA) (Fig. S4†).

**Fig. 2:**
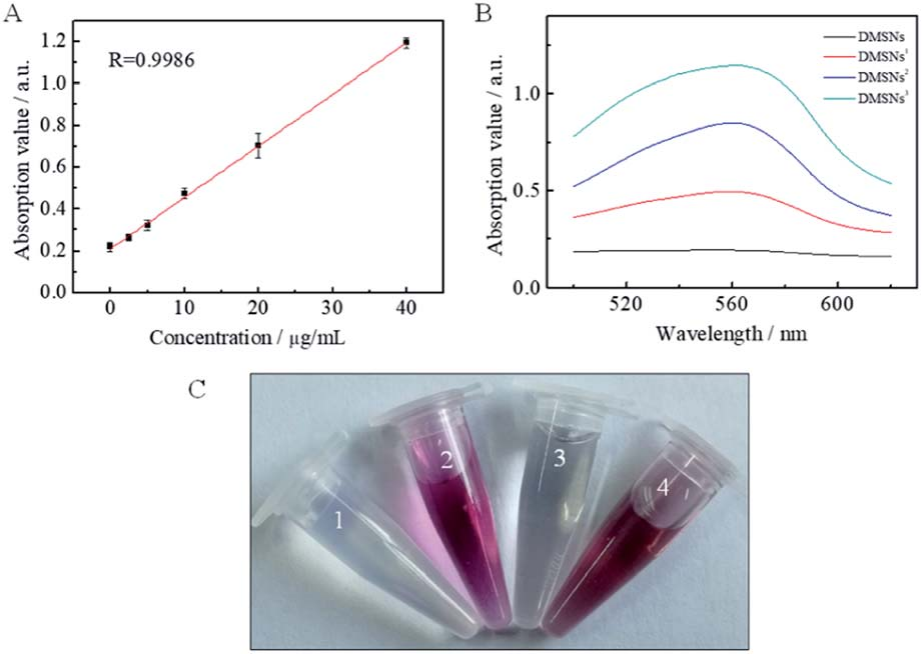
Standard curve of DMSNs^3^@HA (A) and absorption spectra (B) of protein obtained by BCA assay; the carbazole assay to detect HA: (1) DMSNs^3^, (2) HA-NH_2_, (3) The supernate of DMSNs^3^@HA, and (4) DMSNs^3^@HA (C).

Before modifying DMSNs-COOH with an antibody, the role of antibody modification was studied. (1) 0 μg, (2) 12 μg, (3) 24 μg, (4) 36 μg, (5) 48 μg, (6) 60 μg, (7) 72 μg, (8) 84 μg and (9) 96 μg of antibodies were respectively used to modify the DMSNs-COOH by the reaction of amino with carboxyl groups. As shown in Fig. S5A,† the modified antibodies on DMSNs-COOH increased more and more slowly as the total amount of antibodies increased. And the corresponding reaction conversion rate reduced as the antibodies increased (Fig. S5B†), which may be due to the steric hindrance and limited reaction sites. Considering the availability of antibodies, 60 μg of antibodies (12 μg of anti-PD-1, 24 μg of anti-CD3 and 24 μg of anti-CD28) were eventually applied to decorate DMSNs-COOH. ∼7.5 μg of anti-PD-1, ∼8.0 μg of anti-CD3 and ∼8.0 μg of anti-CD28 were eventually modified to 1 mg DMSNs (named DMSNs^3^) by calculating from Fig. 2A and B. The TEM image in showed that DMSNs^3^ showed a uniform dendritic morphology the same as DMSNs. For comparison, DMSNs-COOH modified with only ∼7.5 μg of anti-PD-1 (named DMSNs^1^) or ∼8.0 μg of anti-CD3 and ∼8.0 μg of anti-CD28 (named DMSNs^2^) was also prepared in the same way.

Hyaluronic acid (HA) (structural formula shown in Fig. S6A†) can specifically anchor to the cluster determinant 44 (CD44) receptor that is frequently overexpressed on the surface of tumor cells.^30,31^ Therefore, HA was modified to DMSNs^3^ to target tumor tissue in this work. HA was firstly reacted with ethylenediamine to form HA-NH_2_. Ninhydrin assay data in Fig. S6B† shows bluish violet color for ethylenediamine due to primary amines (1), an achromatic or yellowish color for HA (2), and a dark brown color for HA-NH_2_ due to the primary amines plus amido bonds (CO–NH) (3). The results verified that the HA was successfully modified with amino groups.

Then DMSNs^3^ were modified with HA by the reaction of DMSNs^3^ and HA-NH_2_ to prepare DMSNs^3^@HA. The carbazole assay has been widely applied for the determination of HA in routine analysis.^32,33^ Under acidic conditions, the glucuronic acid resulting from the hydrolysis of HA reacts with carbazole to produce a red-purple substance. As can be seen from Fig. 2C, only (2) HA-NH_2_ and (4) DMSNs^3^@HA changed to red-purple, indicating that HA was successfully modified to DMSNs^3^. The dendritic structure of DMSNs^3^ was still maintained after being modified with HA, which is similar to the shape of dendritic cells (Fig. 1E). Moreover, zeta potential data verified that the nanoparticles were fabricated successfully, *i.e.*, −25.7 ± 0.4 mV (DMSNs), +6.8 ± 0.7 mV (DMSNs-NH_2_), −32.4 ± 1.0 mV (DMSNs-COOH), −14.1 ± 1.2 mV (DMSNs^3^), and −9.4 ± 0.8 mV (DMSNs^3^@HA) (Fig. 1F).

### Biocompatibility of DMSNs^3^@HA

**Fig. 3:**
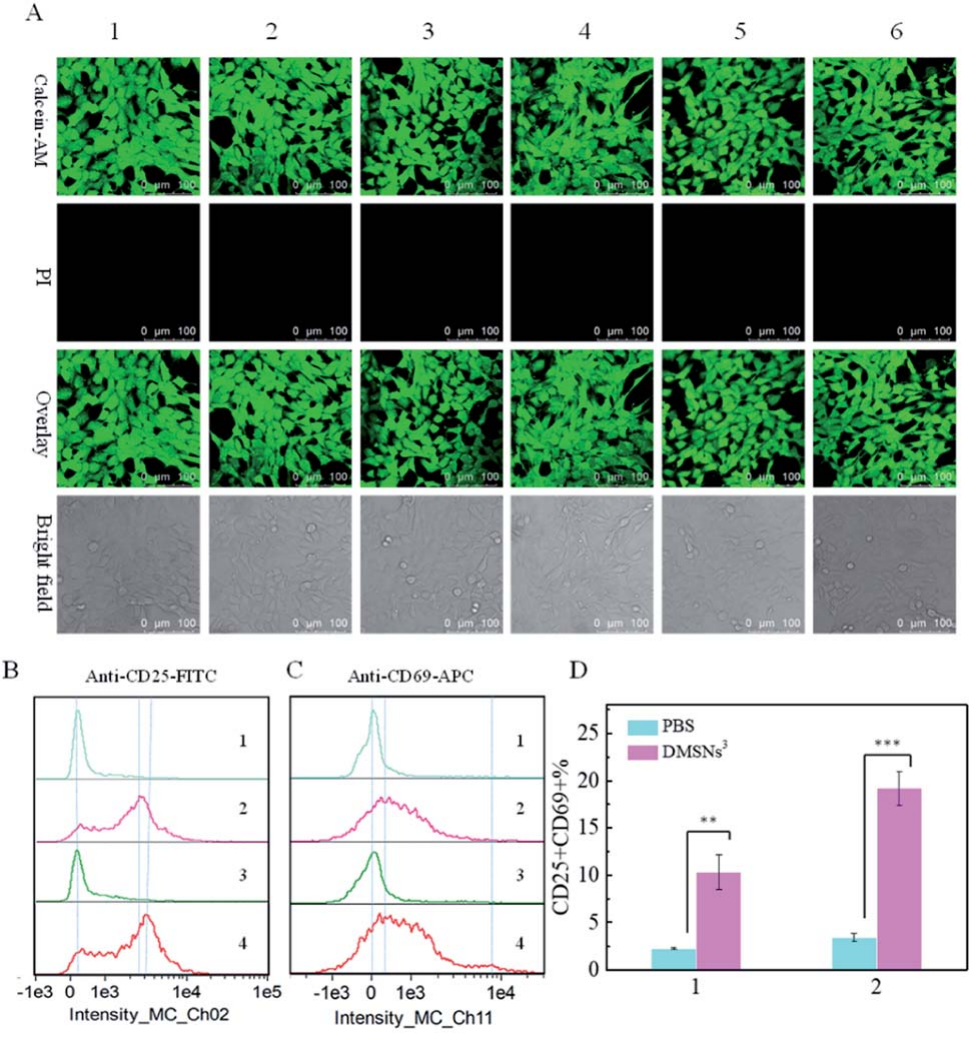
Cell viability obtained by a live/dead assay (A); (1) PBS, (2) DMSNs@HA, (3) DMSNs^1^@HA, (4) DMSNs^2^@HA, (5) DMSNs^3^, and (6) DMSNs^3^@HA (0.2 mg mL^−1^). T cell activation: CD25 (B); CD69 (C); (1) PBS for 4 h; (2) DMSNs^3^ for 4 h (0.2 mg mL^−1^); (3) PBS for 8 h; (4) DMSNs^3^ for 8 h (0.2 mg mL^−1^). The percentage of CD25^+^CD69^+^ T cells: (1) 4 h; (2) 8 h (D).

Given the critical application in biological samples, the biocompatibility of DMSNs^3^@HA was investigated. The viability of 4T1 cells was measured by the live/dead assay and standard MTT assay^34,35^ to evaluate the cytotoxicity of DMSNs^3^@HA. In live/dead assay, calcein-acetoxymethyl ester can be hydrolyzed with intracellular esterases to produce calcein in living cells to show a green fluorescence signal. Propidium iodide can exclusively stain dead cells whose cell membrane is destroyed, which showed red fluorescence. As can be seen from the confocal images in Fig. 3A, all the 4T1 cells in six groups show bright green fluorescence, indicating that the synthesized nanoparticles had good biocompatibility. The data of MTT assay showed that all the viabilities of 4T1 cells were greater than 90%, which also verified the good biocompatibility of DMSNs^3^@HA (Fig. S8†). To further verify the stability, DMSNs^3^@HA particles were immersed in buffer (PBS, Tris and MES), and their hydrodynamic sizes were measured for 7 days by DLS. Fig. S7† shows that the hydrodynamic sizes were constant for 7 days with good repeatability, which indicated that DMSNs^3^@HA presented good stability. The above results revealed that DMSNs^3^@HA has superior biosafety to be applied in the biological field.

### T cell activation

T cells are the critical immune cells in the immune system, whose activation is the key step in the immune cycle. After activation, T cells will express several membrane surface molecules, among which CD69 is the earliest marker of T cell activation.^36–38^ Besides, IL-2R, known as CD25, is also an important marker of T cell activation.^39,40^ Therefore, CD25 and CD69 were detected to verify the activation of T cells in this work. CD8^+^ T cells were harvested by using a MojoSort™ Mouse CD8 T Cell Isolation Kit and incubated with PBS or DMSNs^3^ for different time periods. As shown in Fig. 3B and C, the levels of CD25 and CD69 on CD8^+^ T cells remained basically unchanged after being treated with PBS for 4 h and 8 h, indicating that the T cells were not activated. Importantly, CD8^+^ T cells incubated with DMSNs^3^ demonstrated significantly greater expressions of CD25 and CD69 on T cells, which revealed that the T cells were successfully activated by DMSNs^3^. And the degree of activation increased over time (Fig. 3B and C). In addition, the quantification of CD25^+^CD69^+^CD8^+^ T cells also verified the ability of DMSNs^3^ for activating T cells (Fig. 3D). The activation of CD4^+^ T cells was also verified by using the same method.

Fig. S9† shows that the levels of CD25 and CD69 on CD4^+^ T cells obviously increased after being incubated with DMSNs^3^ for 4 h and 8 h, which was consistent with the above result. The above results demonstrated that our biomimetic nanoparticle can be applicable for activating T cells in biological samples.

### Tumor targeting *in vivo*

**Fig. 4:**
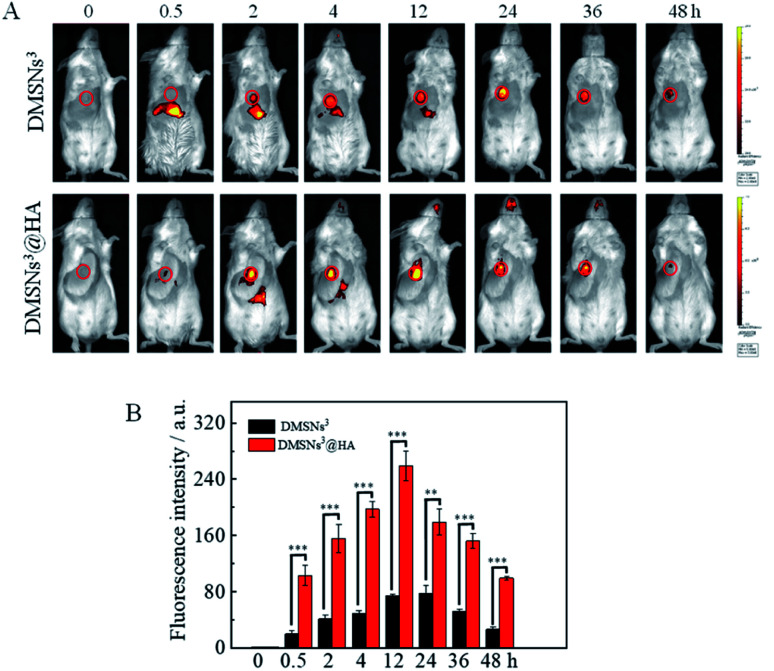
Tumor targeting *in vivo*. Balb/C mice were treated with DMSNs^3^ or DMSNs^3^@HA by intravenous injection and measured at different times (A); the corresponding quantifications of fluorescence intensity of tumors (B). The values are presented as means ± SD. The significance between two groups was analysed by a two-tailed Student's *t*-test (***P <0.001, **P <0.01, *P <0.05, and ^NS^*P* >0.05).

HA has frequently been utilized as a ligand to target cancer cells because the hyaluronic acid (HA) receptor, CD44, is overexpressed on the surface of cancer cells. Inspired by this, HA was immobilization to DMSNs^3^ to obtain the “smart bullet” DMSNs^3^@HA to specifically target the tumor and reduce the side effect. To study the tumor targeting ability of DMSNs^3^@HA, a near infrared fluorescent dye (IR 808) was loaded in this biomimetic nanoparticle. After being treated with DMSNs^3^ or DMSNs^3^@HA, the fluorescence signals at tumors (circle) first increased and then decreased as time goes on by using an *in vivo* imaging system (Fig. 4A). Moreover, DMSNs^3^@HA showed gradually increased tumoral fluorescence intensity and the fluorescence intensity was much higher than that with DMSNs^3^ (Fig. 4B), which demonstrated that the “smart bullet” DMSNs^3^@HA has better ability for targeting tumor tissues.

### Therapeutic effects *in vivo*

**Fig. 5:**
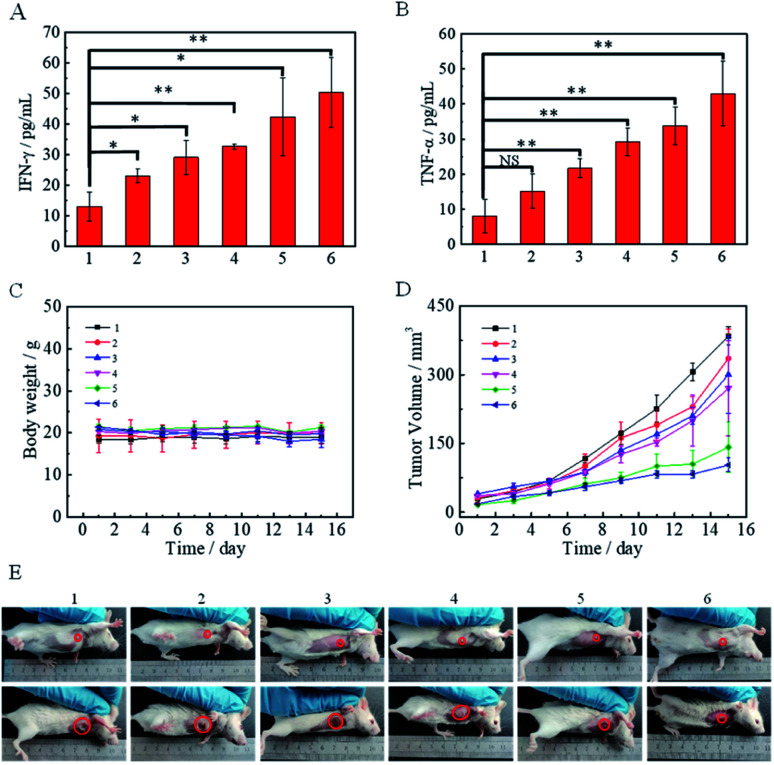
Therapeutic effects *in vivo*. IFN-γ (A) and TNF-α (B) in serum after different treatments; the changes of tumor growth (C), weight of mice (D) and pictures of tumors (E) after the treatments of Balb/C mice: (1) PBS, (2) DMSNs@HA, (3) DMSNs^1^@HA, (4) DMSNs^2^@HA, (5) DMSNs^3^, and (6) DMSNs^3^@HA. The values are presented as means ± SD. The significance between two groups was analysed by a two-tailed Student's *t*-test (***P <0.001, **P <0.01, *P <0.05, and ^NS^*P* >0.05).

Interferon γ (IFN-γ), which is produced by CD8^+^ T cells, plays a vital role in immunomodulatory and anti-tumor activity. Moreover, IFN-γ has effects on the release of tumor necrosisfactor-α (TNF-α), and synergizes with each other to function in the immune system.^41–43^ Therefore, TNF-α and IFN-γ in the serum of mice after various treatments: (1) PBS, (2) DMSNs@HA, (3) DMSNs^1^@HA, (4) DMSNs^2^@HA, (5) DMSNs^3^, and (6) DMSNs^3^@HA were measured to verify the activated immune response. Fig. 5A and B show that the group 6 DMSNs^3^@HA expresses more IFN-γ and TNF-α, which indicated that the immune response was successfully triggered by our biomimetic nanoparticle *in vivo*. Too many cytokines may cause damage to the vital organs. Therefore, hematoxylin-eosin (H&E) staining was performed on the heart, liver, spleen, kidney and lung of mice to study the safety of the materials for normal tissues. H&E staining of the five major organs showed that no tissue of mice was damaged after various treatments (Fig. S11†), which verified the biosecurity of our biomimetic nanoparticles. Inspired by this, the antitumor performance of DMSNs^3^@HA was evaluated on 4T1 tumor-bearing Balb/C mice. Mice were randomly divided into 6 groups and intravenously administrated with: (1) PBS, (2) DMSNs@HA, (3) DMSNs^1^@HA, (4) DMSNs^2^@HA, (5) DMSNs^3^, and (6) DMSNs^3^@HA (50 mg kg^−1^) 5 times in total. The tumor volumes and body weights were measured every other day (Fig. 5C and D). As can be seen from Fig. 5C, the group 2 DMSNs@HA suppressed the growth of tumors to a certain degree, which may be because of the immunoadjuvant activity of DMSNs.^44,45^ In group 3, DMSNs^1^@HA showed only limited advantages compared with DMSNs@HA, which was due to the immunosuppression of breast cancer. Tumors of mice were gained and studied by immunofluorescence staining. Fig. S10† shows that DMSNs^3^@HA groups exhibited the most CD4^+^ and CD8^+^ T cells in tumor slides, which indicated the most significant immune response in accordance with the ELISA results. DMSNs^3^ presented a better antitumor ability than DMSNs^1^@HA and DMSNs^2^@HA, indicating that the T cell activation combining immune checkpoint blocking induced the “1 + 1 >2” effect of immunotherapy. Significantly, DMSNs^3^@HA exhibited the best therapeutic effect among the 6 groups, which revealed that our biomimetic nanoparticle had superior performance in modulating immune response for cancer therapy (Fig. 5D and E and S12†).

## Conclusions

In summary, we have presented a dendritic cell-like biomimetic nanoparticle (DMSNs^3^@HA) that can specifically target and activate T cells at tumor sites for treating immunosuppressive tumors. This biomimetic nanoparticle has the following advantages: (1) accurate targeting of the tumor by modification of HA; (2) activation of T cells by anti-CD3 and anti-CD28 just like DCs; (3) breaking of the immune “brake” of T cells to further promote the activation of T cells by blocking PD-1/PD-L1. Flow cytometry data showed that our DMSNs^3^@HA can effectively activate T cells and improve their immune response. In the 4T1 mouse model, DMSNs^3^@HA can significantly improve the therapeutic effect of breast cancer by activating T cells and breaking the immune “brake” compared with checkpoint blockade exclusively (DMSNs^1^@HA treatment group). Moreover, it also proved that T cell activation combining immune checkpoint blocking induced the “1 + 1 >2” immunotherapy effect against immunosuppressive tumors. We expect that this strategy will provide new insights into tumor immunotherapy by modulating T cell behavior.

## Ethical statement

Animal experiments were reviewed and approved by the Ethics Committee of Shandong Normal University, Jinan, P. R. China (approval number AEECSDNU 2019033). All the animal experiments complied with the relevant guidelines of the Chinese government and regulations for the care and use of experimental animals.

## Data availability

All experimental data and procedures are provided in the ESI.†

## Author contributions

Y. L., N. L. and B. T. conceived and designed the experiments. Y. L. and K. T. performed the experiments. K. T., X. Z., W. P., N. L. and B. T. analyzed the data. Y. L. and K. T. contributed the schematic materials. Y. L., K. T., N. L. and B. T. co-wrote the paper. X. Z., W. P., N. L. and B. T. edited the manuscript.

## Conflicts of interest

The authors declare no competing financial interest.

## Supplementary Material

SC-013-D1SC03525H-s001
