# Supplementary material for: A dendritic cell-like biomimetic nanoparticle enhances T cell activation for breast cancer immunotherapy
Source: Chem Sci. 2021 Nov 25;13(1):105–10. doi: 10.1039/d1sc03525h (PMC8694320; doi:10.1039/d1sc03525h)
Supplement: SC-013-D1SC03525H-s001 [file SC-013-D1SC03525H-s001.pdf]

Electronic Supplementary Material (ESI) for Chemical Science.
This journal is © The Royal Society of Chemistry 2021

A dendritic cell-like biomimetic nanoparticle enhances T cell activation for breast cancer immunotherapy

Yanhua Li,‡ Kun Tang,‡ Xia Zhang, Wei Pan, Na Li* and Bo Tang*

Experimental Procedures

Reagents and Instruments. InVivoMAb anti-mouse PD-1 (CD279) (150 KDa), InVivoMAb anti-mouse CD3 (150 KDa), InVivoMAb anti-mouse CD28 (150 KDa) were purchased from Bio X Cell, USA. MojoSort™ Mouse CD8 T Cell Isolation Kit, APC anti-mouse CD69 Antibody and FITC anti-mouse CD25 Antibody were purchased from BioLegend, Inc. Cetyltrimethylammonium tosylate (CTATos) was purchased from Sigma, Triethanolamine ($TEAH_3$), tetraethylorthosilicate (TEOS), (3-aminopropyl) trimethoxysilane (APTMS), succinic anhydride, ninhydrin, ethylenediamine, 1-butyl-3-methylimidazolium trifluoro-methanesulfonate ([BMIM] OTF), N,N-Dimethylformamide (DMF), carbazole, H_2SO_4 were purchased from China National Pharmaceutical Group Corporation, China. Fetal bovine serum (FBS) was purchased from Gibco, USA. Trypsin-EDTA was purchased from Gibco, USA. Live & Dead Viability/Cytotoxicity Assay Kit for Animal Cells and RPMI 1640 medium was purchased from Key Gen Biotech. Co., Ltd. The BCA Protein Assay Kit, Mouse IFN-γ Mini ELISA Kit and Mouse TNF-α ELISA Kit were purchased from Boster Biological Technology Co., Ltd, China. 3-(4,5)-dimethylthiahiazo(-z-y1)-3,5-di-phenytetrazoliumromide (MTT) was purchased from Beijing Solarbio Science &

Technology Co., Ltd. Glass Bottom dishes were purchased from Cellvis, Mountain View, CA. 96-well plates were purchased from Hangzhou Xinyou Biotechnology Co., Ltd, China. Plastic centrifuge tubes were purchased from GeneBrick Bioscience LLC. Red blood cell lysis buffer was purchased from Sangon Biotech Co., Ltd. All the aqueous solutions used in experiments were prepared using deionized water (18.2 MΩ cm) obtained from a Milli-Q water purification system. All chemicals were of analytical grade and were used without further purification. Transmission electron microscopy (TEM) was carried out on a HT7700 electron microscope (Hitachi, Japan). The *in vivo* imaging study was performed with a Caliper IVIS Lumina III imaging system (Caliper Co., USA). The flow cytometer assays were carried out with an imaging flow cytometer (Amnis Corporation, USA), and IDEAS image analysis software (Amnis) was used to analyze the images.

Cell lines and animals. 4T1 cells were cultured in RPMI 1640 medium containing 10% FBS and 100 U mL^{-1} of 1% antibiotics (penicillin/streptomycin) and were maintained at 37 °C in a 5% CO_2/95% air humidified incubator (SANYO). Animal experiments were reviewed and approved by the Ethics Committee of Shandong Normal University, Jinan, P. R. China (approval number AEECSDNU 2019033). All the animal experiments complied with relevant guidelines of the Chinese government and regulations for the care and use of experimental animals.

Synthesis of the dendritic mesoporous silica nanoparticles (DMSNs). 0.96 g of CTATos, 0.105 g of $TEAH_3$, 0.01g of [BMIM] OTF and 50 mL of H_2O were mixed under 80 °C for at least 30 min. Then 7.86 mL of TEOS was added into the above solution

quickly and stirred for another 2 h. The obtained product was centrifuged and stirred in the saturated NaCl/MeOH for at least 48 h to remove the template. After that, the synthesized DMSNs were washed by ethanol and water for three times, and then dried for later use.

Synthesis of the DMSNs modified with amino and carboxyl groups. 40 mg of the prepared DMSNs, 4 mL of H_2O and 10 mL of ethanol were homogeneously mixed. Then 100 μL of ammonium hydroxide was added to the above solution to adjust the pH. After 30 min, 40 μL of APTMS was added and stirred overnight to modify the DMSNs with amino groups (DMSNs-NH_2). The DMSNs-NH_2 were centrifuged and washed by ethanol and water for three times. Ninhydrin assay was carried out to verify the modification of amino groups on DMSNs-NH_2. 1 % of ninhydrin and DMSNs-NH_2 were mixed and subjected to boiling. That ninhydrin reacted with supernatant liquid of the centrifuged DMSNs-NH_2 solution served as the control group.

DMSNs-NH_2 were dispersed in anhydrous DMF, following with addition of excess succinic anhydride. The reaction continues overnight to obtain the DMSNs modified with carboxyl groups (DMSNs-COOH). The product were centrifuged and washed by ethanol and water for three times.

Synthesis of the DMSNs modified with antibodies. 1 mg of DMSNs-COOH were activated by 1.5 mg of EDC and 0.7 mg of NHS for 30 min. 12 μg of anti-PD-1 was added to the solution and stirred for 10 min. And then 24 μg of anti-CD3 and 24 μg of anti-CD28 were added to the above solution and stirred overnight at 4 ℃ to react

with the activated DMSNs-COOH. DMSNs-COOH modified with only anti-PD-1 were named as DMSNs^{1}. DMSNs-COOH modified with anti-CD3 and anti-CD28 were named as DMSNs^{2}. DMSNs-COOH modified with anti-PD-1, anti-CD3 and anti-CD28 were named as DMSNs^{3}.

Synthesis of the HA modified with amino groups. 1 g of HA monomer was dispersed in pH 6.0 MES buffer. And then 2.5 g EDC and 1.5 g NHS were added to the solution and stirred for 30 min to activate carboxyl groups. After that, 4.75 g of ethylenediamine was added and stirred overnight. The product (HA-NH_2) was dialysed by dialysis membrane (300 Da) and freeze-dried for further use. Ninhydrin assays were carried out to verify the modification of amino groups on HA-NH_2. Ethylenediamine, HA and HA-NH_2 were respectively mixed with 1 % of ninhydrin and subjected to boiling to observe the change of color.

Synthesis of the DMSNs^{3} modified with HA. 1 mg of DMSNs^{3} was dispersed in pH 6.0 MES buffer with addition of 2 mg of EDC and 1 mg of NHS for 30 min. And then 7 mg of HA-NH_2 was added and the reaction continued overnight to obtain DMSNs^{3}@HA at 4 ^{o}C.

T cell activation. CD8^{+} T cells were harvested by MojoSort™ Mouse CD8 T Cell Isolation Kit. Spleens were extracted from healthy mice, and single-cell suspension was prepared and dispersed in the cold buffer. After filtered by filter membrane (70 μm), the cell density was adjusted to 10^{8} /mL. 10 μL of biotin-Antibody Cocktail was added into 100 μL of above cells. Mix and incubate on ice for 15 min. Then 10 μL of streptavidin was added and incubate on ice for another 15 min. After added with 2.5

mL of buffer, the mixed cell solution was placed in the magnet for 5 min. Collect the supernate and repeat the separation steps to obtain the CD8+ T cells with high purity for further use.

$CD8^+$ T cells were divided into two groups and incubated with: 1) PBS; 2) 0.2 mg/mL $DMSNs^3$ for different time. Then the T cells were stained with anti-CD69-APC and anti-CD25-FITC and detected by flow cytometry to verify the activation of T cells.

$CD4^+$ T cells were harvested by MojoSort™ Mouse CD4 T Cell Isolation Kit. The other steps were the same as above.

***In vivo* experiments.** For the xenografts established from cultured cells00, 4T1 cells were suspended and harvested after trypsinization, and approximately 5×10^5 4T1 cells in 150 μL of serum-free RPMI 1640 medium were subcutaneously injected into the right flank of the mice. The tumor volume (V) was determined by measuring the length (L) and width (W) and was calculated as $L \times W^2/2$.

To study the antitumor efficacy, mice with tumors were randomly divided into six groups and subjected to different treatments: 1), PBS; 2), DMSNs@HA; 3), $DMSNs^1$@HA; 4), $DMSNs^2$@HA; 5), $DMSNs^3$; and 6), $DMSNs^3$@HA. The intravenously injected dose was 50 mg/kg each time for 5 times in total. The tumor volumes and body weights of the mice were measured every other day for 14 days. Haematoxylin and eosin (H&E) staining of the five major organs (heart, liver, spleen, lung and kidney), routine blood tests and blood biochemical tests were carried out at 24 h post-treatment to prove that the body's immune response resulted in minimal side effects to major organs.

The carbazole assay. The samples were placed in the ice water bath. Gradually add in 5 mL of pre-cooling $Na_2B_4O_7/H_2SO_4$ (0.025 M) while stirring in the ice water bath. Then the mixed solution was kept in boiling water for 20 min. Cool to room temperature and add in 0.2 mL of carbazole/ethanol (0.1%) to observe the color change after 2 h.

***In vivo* imaging.** The fluorescence images were taken by an *in vivo* imaging system (IVIS). The Living Image software (Xenogen) was used to acquire the data at different times (0, 0.5, 2, 4, 12, 24, 36, 48 h) after treatments.

ELISA analysis. Serum cytokine levels were determined by enzyme-linked immunosorbent assays (ELISAs) using antibody pairs specific to these cytokines, following protocols recommended by the manufacturer. Mice with tumors were divided into six groups (n = 6) and subjected to different treatments for five times: 1), PBS; 2), DMSNs@HA; 3), $DMSNs^1$@HA; 4), $DMSNs^2$@HA; 5), $DMSNs^3$; and 6), $DMSNs^3$@HA. After 12 h, the mice were sacrificed to harvest serum. Serum levels of IFN-γ and TNF-α were determined with the Mouse IFN-γ Mini ELISA Kit and Mouse TNF-α ELISA Kit, respectively.

Supporting Figures

Fig. S1 Particle size of DMSNs as determined by dynamic light scattering (DLS).

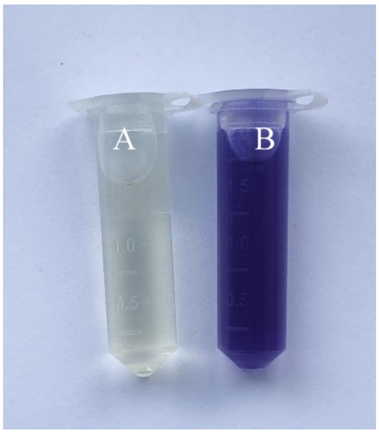

Fig. S2 Ninhydrin assays to verify the amino groups: supernatant liquid of the centrifuged DMSNs-NH_2 (A); the precipitate of DMSNs-NH_2 (B).

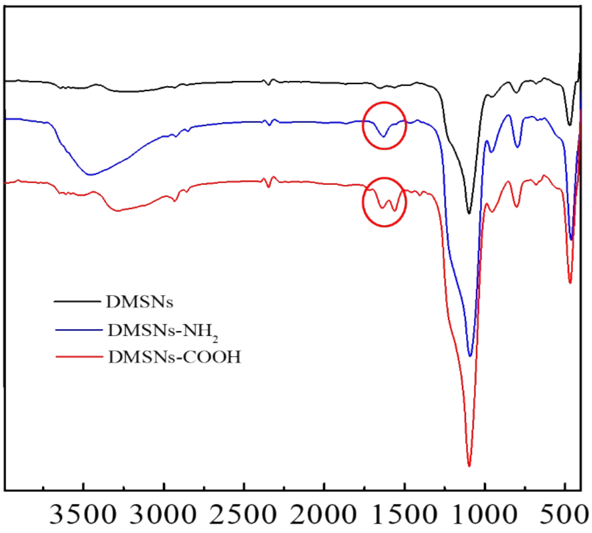

Fig. S3 The FT-IR of DMSNs (Black); DMSNs-NH_2 (Blue); DMSNs-COOH (Red).

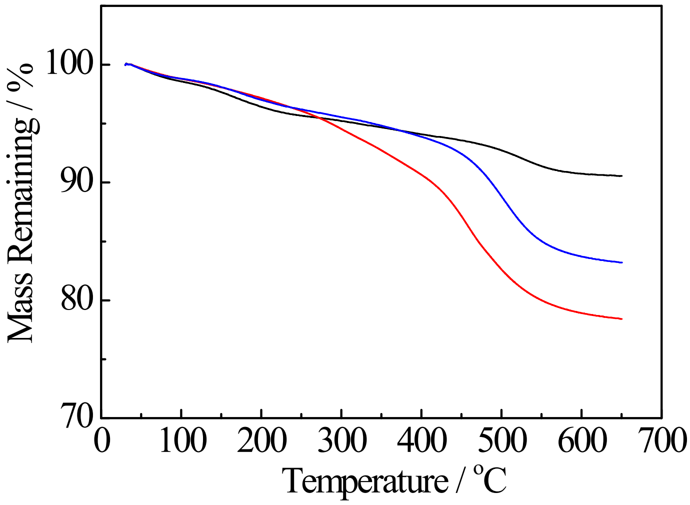

Fig. S4 TGA of DMSNs (Black); DMSNs-NH_2 (Blue); DMSNs-COOH (Red).

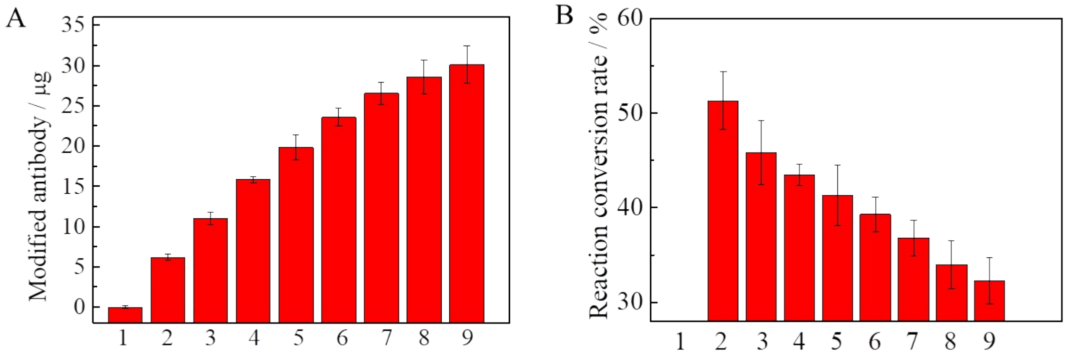

Fig. S5 Modified antibody on DMSNs (A); The reaction conversion rate of antibody on DMSNs (B); Total antibody: 1) 0 μg, 2) 12 μg, 3) 24 μg, 4) 36 μg, 5) 48 μg, 6) 60 μg, 7) 72 μg, 8) 84 μg and 9) 96 μg

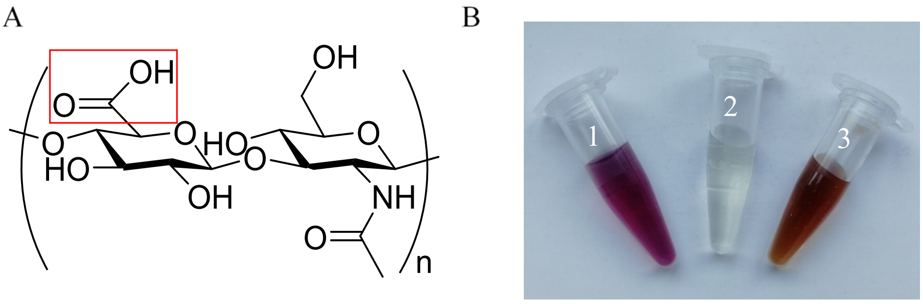

Fig. S6 Structural formula of HA (A); Ninhydrin assays to verify the modification of HA: Ethylenediamine (1), HA (2) and HA-NH_2 (3) (B).

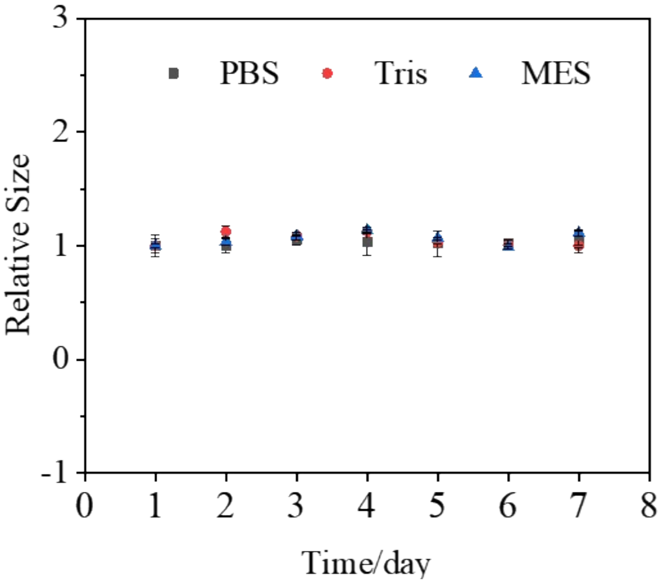

Fig. S7 Stability of DMSNs^3@HA in buffer by DLS.

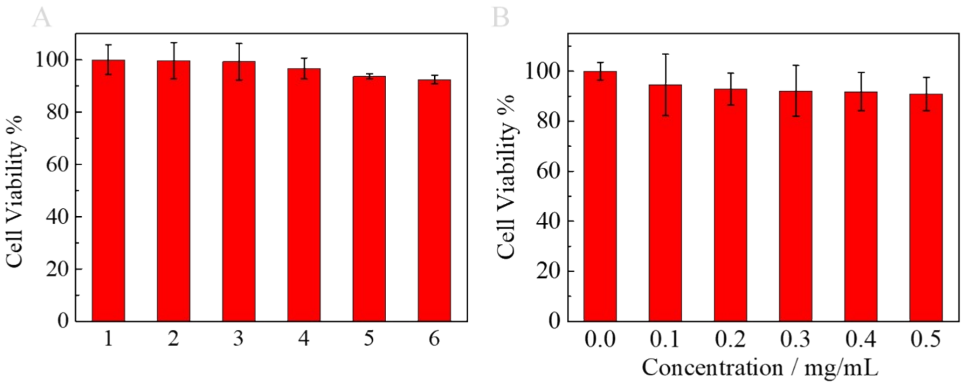

Fig. S8 Cell viability of 4T1 cells incubated with 1), PBS, 2), DMSNs@HA, 3), DMSNs^1@HA, 4), DMSNs^2@HA, 5), DMSNs^3, and 6), DMSNs^3@HA (0.2 mg/mL) (A). Cell viability of 4T1 cells incubated with DMSNs^3@HA (0-0.5 mg/mL) (B).

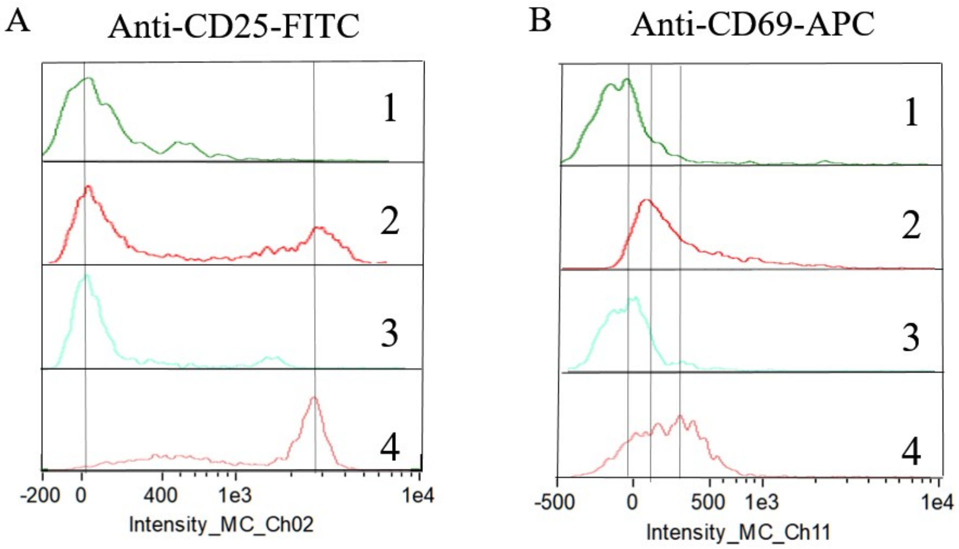

Fig. S9 T cell activation by flow cytometry: CD25 (A); CD69 (B); 1) PBS for 4 h; 2) DMSNs^3 for 4 h (0.2 mg/mL); 3) PBS for 8 h; 4) DMSNs^3 for 8 h (0.2 mg/mL).

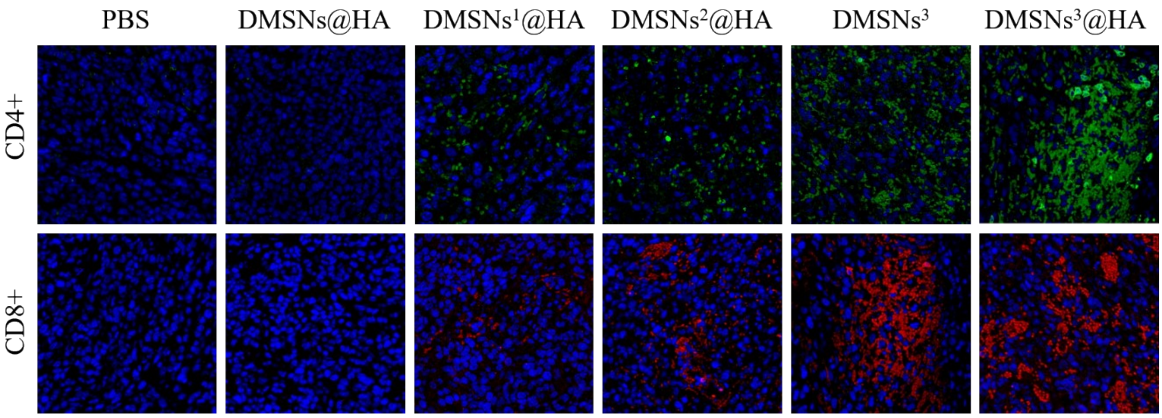

Fig. S10 Immunofluorescence staining assay of $CD4^+$ T cells and $CD8^+$ T cells.

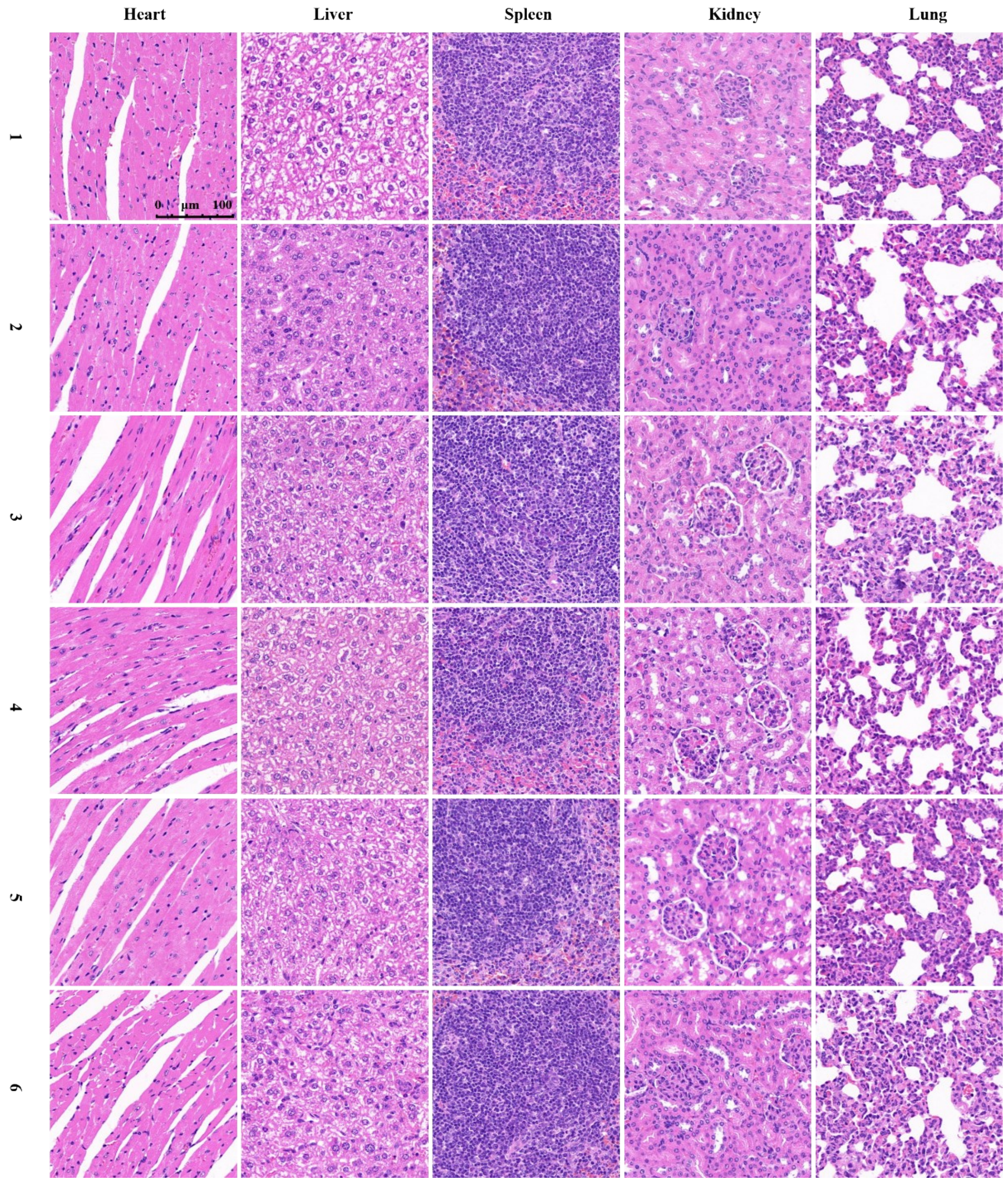

Fig. S11 H&E staining of the five major organs (heart, liver, spleen, kidney and lung after different treatments: 1), PBS, 2), DMSNs@HA, 3), DMSNs[1]@HA, 4), DMSNs[2]@HA, 5), DMSNs[3], and 6), DMSNs[3]@HA.

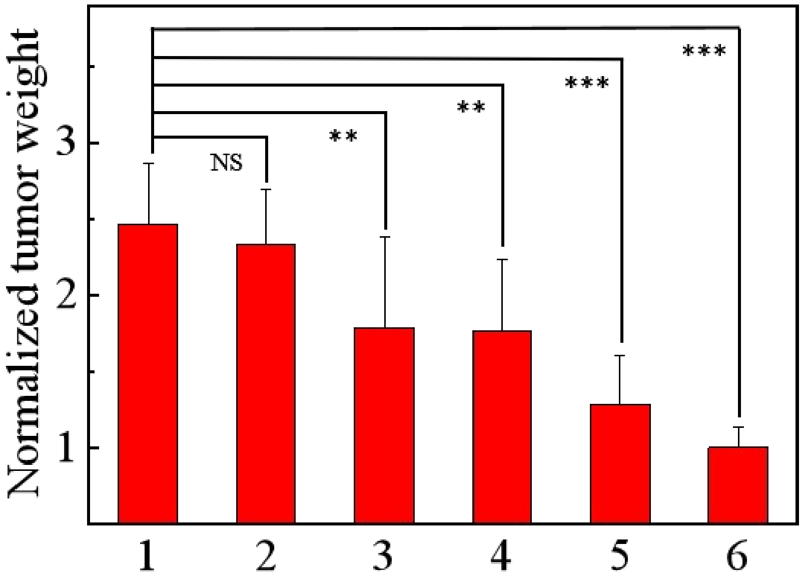

Fig. S12 The normalized tumor weight after different treatments: 1), PBS, 2), DMSNs@HA, 3), DMSNs[1]@HA, 4), DMSNs[2]@HA, 5), DMSNs[3], and 6), DMSNs[3]@HA. The values are presented as means ± SD. The significance between two groups was analysed by a two-tailed Student t-test (***P<0.001, **P<0.01, *P<0.05, ^{NS}P>0.05).
